# Diagnostic Accuracy of Arterial Spin Labeling in Comparison With Dynamic Susceptibility Contrast-Enhanced Perfusion for Brain Tumor Surveillance at 3T MRI

**DOI:** 10.3389/fonc.2022.849657

**Published:** 2022-05-20

**Authors:** Anna Lavrova, Wouter H. T. Teunissen, Esther A. H. Warnert, Martin van den Bent, Marion Smits

**Affiliations:** ^1^ Department of Radiology, University of Michigan Hospital, Ann Arbor, MI, United States; ^2^ Department of Radiology & Nuclear Medicine, Erasmus MC, Rotterdam, Netherlands; ^3^ Brain Tumour Centre, Erasmus MC Cancer Institute, Rotterdam, Netherlands; ^4^ Department of Neurology, Erasmus MC, Rotterdam, Netherlands

**Keywords:** MRI, DSC, brain tumor, glioma, brain metastasis, perfusion MRI, ASL, diagnostic accuracy

## Abstract

**Purpose:**

We aimed to compare arterial spin labeling (ASL) with dynamic susceptibility contrast (DSC) enhanced perfusion MRI for the surveillance of primary and metastatic brain tumors at 3T, both in terms of lesion perfusion metrics and diagnostic accuracy.

**Methods:**

In this retrospective study, we included 115 patients, who underwent both ASL and DSC perfusion in the same 3T MRI scanning session between 1 January and 31 December 2019. ASL-derived cerebral blood flow (CBF) maps and DSC-derived relative cerebral blood volume (rCBV) maps, both uncorrected and corrected for leakage, were created with commercially available software. Lesions were identified as T2-/T2-FLAIR hyperintensity with or without contrast enhancement. Measurements were done by placing a region of interest in the visually determined area of highest perfusion, copying to the contralateral normal appearing white matter (NAWM), and then propagating to the other perfusion maps. Pearson’s correlation coefficients were calculated between the CBF and rCBV ratios of tumor versus NAWM. Accuracy for diagnosing tumor progression was calculated as the area under the receiver operating characteristics (ROC) curve (AUC) for the ASL-CBF and leakage corrected DSC-rCBV ratios.

**Results:**

We identified 178 lesions, 119 with and 59 without contrast enhancement. Correlation coefficients between ASL-derived CBF versus DSC-derived rCBV ratios were 0.60–0.67 without and 0.72–0.78 with leakage correction in all lesions (n = 178); these were 0.65–0.80 in enhancing glioma (n = 80), 0.58–0.73 in non-enhancing glioma, and 0.14–0.40 in enhancing metastasis (n = 31). No significant correlation was found in enhancing (n = 8) or non-enhancing (n = 7) lymphomas. The areas under the ROC curves (AUCs) for all patients were similar for ASL and DSC (0.73–0.78), and were higher for enhancing glioma (AUC = 0.78–0.80) than for non-enhancing glioma (AUC = 0.56–0.62). In brain metastasis, the AUC was lower for ASL-derived CBF (AUC = 0.72) than for DSC-derived rCBV ratios (AUC = 0.87–0.93).

**Conclusion:**

We found that ASL and DSC have more or less the same diagnostic accuracy. Our findings suggest that ASL can be used as an alternative to DSC to measure perfusion in enhancing and non-enhancing gliomas and brain metastasis at 3T. For lymphoma, this should be further investigated in a larger population.

## Introduction

The global incidence of brain tumors, including brain metastasis, has increased over the past 20 years by more than 40%, and current estimates assume 3.4 patients with a brain tumor per 100,000 people. Once diagnosed with a brain tumor, most patients have a poor prognosis, especially in the elderly (>65 years) (1). Gliomas are the most frequent cause (>80%) of a primary intra-axial brain tumor, with glioblastoma being the most frequent subgroup (45%) and associated with very limited survival (5% 5-year survival) (2). However, brain metastases are the most common intracranial intra-axial tumors overall, occurring in 15–40% of all cancer patients (3). The most frequent primary sites of spread to the brain are the lung (41–56%), breast (13–30%), and melanoma (6–11%) (4).

In the past two decades, most primary and secondary brain tumor treatments have been improved, requiring the monitoring of the cerebral disease activity in most patients. Currently, magnetic resonance imaging (MRI) is the method of choice for diagnosis and surveillance of brain tumors, and the administration of a gadolinium-based contrast agent (GBCA) allows the identification of blood–brain barrier (BBB) disruption in the form of contrast enhancement. Contrast enhancement is considered a surrogate marker of tumor activity but remains a nonspecific finding and may also occur in relation to treatment effects. This can complicate the assessment of treatment response (5–7). For both irradiated treated glioma and metastasis treated with focal (high dose) stereotactic radiation therapy, the distinction between tumor progression and treatment effects (so-called pseudoprogression) can be challenging using conventional MRI only, as both progression and pseudoprogression cause BBB impairment (8, 9). In these cases, advanced MR-techniques, such as perfusion MRI, could overcome the limitations of conventional MRI.

Dynamic susceptibility contrast (DSC) MRI is an established perfusion MRI method that allows estimation of the tissue vascularization through measurements of cerebral blood volume (CBV) and cerebral blood flow (CBF) in the suspicious lesion (10, 11). This technique—based on T2 or T2* weighted imaging—estimates perfusion using an exogenous GBCA, which allows for differentiating pseudoprogression from disease progression, especially in high-grade tumors (e.g., glioblastoma) (12). It has also been used to distinguish low-grade from high-grade tumors and for glioma grading (13, 14). Probably the most important issue with DSC is related to its artifacts, including the “leakage” of contrast bolus into the extravascular space, with as a consequence the underestimation of the true CBV due to T1-relaxation effects; and the susceptibility artifacts, which appear at the skull base, paranasal sinuses, or at the site of resection cavity due to post-treatment hemorrhage or metallic surgical material (10). The former issue is commonly addressed by adding a preload GBCA bolus along with leakage correction post-processing tools (15, 16). Additionally, GBCA use is contraindicated in patients with hypersensitivity reactions to gadolinium and in patients with renal insufficiency (17, 18). It can also be challenging to administer GBCA due to traumatic intravenous cannulation in patients with fragile veins, e.g., elderly people or intravenous chemotherapy recipients (19). Also, recent scientific data indicate an unfavorable gadolinium deposition in various organs and tissues after repetitive GBCA administrations, which could lead to toxicity (20).

Arterial spin labeling (ASL) is another perfusion MRI method that provides an alternative that overcomes some of the DSC-MRI related issues. It does not require contrast media administration and provides a quantification of CBF using magnetically labeled blood water as an endogenous tracer. Importantly, ASL derived CBF measurements are insensitive to BBB disruption because blood water is a freely diffusible tracer. Also, the technique is currently available on scanners from all major vendors with 3D acquisition schemes that are minimally affected by susceptibility artifacts. Although the principle of ASL was first implemented in the 1990s (21), the interest in this technique has increased rapidly within the past decade due to improving MRI systems and pulse sequences and concerns with GBCA administration (22). Several studies have been conducted with the use of ASL compared with DSC-MRI in brain tumor patients (12, 14, 23), showing a close correlation between these methods. These studies were generally small, performed in patients with glioma only and compared primarily CBV derived from DSC-MRI with CBF derived from ASL, without considering the diagnostic accuracy. Furthermore, the impact of the chosen software package and post-processing method was not taken into account. No large comparative studies in patients with brain metastasis exist.

In our study, we present a comparative analysis of DSC-MRI and ASL in the surveillance of patients with primary and metastatic brain tumors scanned at 3T. The aim was to determine whether ASL is comparable to DSC in daily clinical practice in terms of correlation between measurements and their diagnostic accuracy. To assess the impact of post-processing, we used two commercially available DSC-MRI analysis software packages and performed analyses both with and without leakage correction.

## Materials and Methods

### Study Design

In this single-center retrospective study, a cohort of consecutive patients was included, who were under surveillance for any intra-axial brain tumor and scanned between 1 January and 31 December 2019 at 3T MRI at the Erasmus MC, Rotterdam, the Netherlands. This cohort constitutes a small proportion of all patients scanned at our institution for this indication, most of whom are scanned at 1.5T. Patients were randomly scheduled for scanning at 1.5T or 3T, i.e., there was no selection bias. Only patients who were scheduled at 3T underwent both DSC-MRI and ASL as part of their routinely performed surveillance scan, and all these patients were enrolled in this study. Clinical information was obtained from the electronic health records and consisted of information on general demographics, clinical diagnosis, and histopathology. Follow-up (for a minimum of three months) data were used to confirm the clinical diagnosis in case no histopathology was available. Both radiological and clinical information were used to define tumor progression, pseudoprogression, stable disease, or response in line with the criteria formulated by Ellingson et al. (24). This study was reviewed by the Erasmus MC Medical Ethics Committee (MEC-2020-0267) and conducted according to the Declaration of Helsinki.

### MRI Acquisition and Post-Processing

MRI scans were performed on two 3T MRI scanners (GE Healthcare, Milwaukee, IL, USA) using a 32- or 48-channel head coil. The conventional MRI protocol consisted of pre- and post-contrast 3D T1w FSPGR, DWI, 3D T2w-FLAIR, and T2w. ASL was acquired as a 3D pseudocontinuous sequence with spiral readout and background suppression using flip angle (FA) = 111°, echo time (TE) = 10.6 ms, repetition time (TR) = 4,635 ms, label duration of 4 s and a single post-labeling delay (1.5 s); the reconstructed voxel size was 1.9 × 1.9 × 3.5 mm^3^. DSC was performed approximately 5 min after a full-dose preload bolus (0.1 mmol/kg Gadovist^®^ 1.0, Bayer Pharmaceuticals, Germany), with a second full-dose bolus of GBCA injected during a 2D gradient echo EPI acquisition using FA = 90°, TE = 45 ms, TR = 2 s, number of volumes = 52, voxel size = 2.0 × 2.0 × 5.0 mm^3^.

ASL-derived CBF maps were created with Ready View (AW Server, GE, USA). For DSC post-processing, two software packages were used: Intellispace Portal (ISP) (Philips Healthcare, The Netherlands) and IB Neuro (IBN) (Imaging Biometrics, USA). Even though the recommended standard is to apply leakage correction, DSC-derived relative cerebral blood volume (rCBV) maps were created both with and without leakage correction, to assess the impact that leakage correction has on the correlation with ASL-derived CBF from a methodological perspective. Hence, 4 rCBV maps were derived from the DSC-MRI scan of each patient: ISP leakage uncorrected, ISP leakage corrected, IBN leakage uncorrected, and IBN leakage corrected.

### Image Analysis

First, a visual assessment of the image quality was performed. The parameters included in the evaluation were scored in a dedicated electronic case record form, and consisted of the overall quality of the scan, the presence of artifacts (e.g., motion, susceptibility) either in general or at the site of the lesion (considered lesion specific issues), and contrast enhancement on post-contrast T1w imaging.

**Figure 1:**
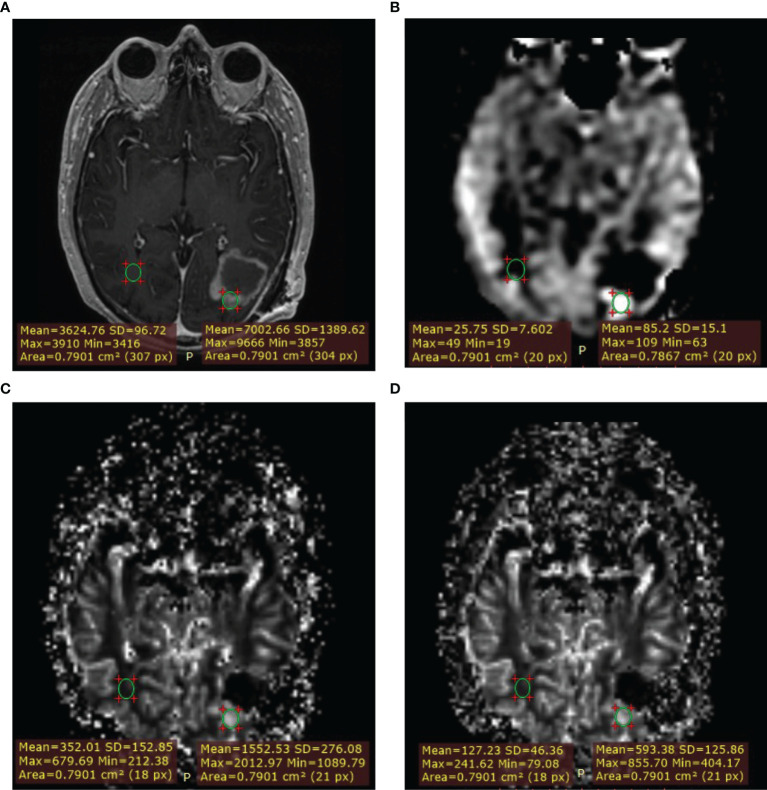
Example of measurement with region of interest (ROI) placement. Lesion ROI and contralateral normal appearing white matter (NAWM) ROI. **(A)** T1W+Gd. **(B)** ASL-CBF. **(C)** DSC-rCBV. **(D)** DSC-rCBV leakage corrected.

Second, a quantitative approach was taken. Lesions were identified on post-contrast T1w or T2w/T2w-FLAIR images if contrast enhancement was absent. Measurements were done in Radiant DICOM Viewer by placing a region of interest (ROI) of approximately 70 mm^2^ (mean 78 mm^2^) in a representative part of the lesion with the visually highest perfusion (‘hot spot’) on the DSC derived ISP leakage corrected rCBV map and copying it to the contralateral normal appearing white matter (NAWM) on the same image slice. For anatomical reference, overlays of the perfusion map on the post-contrast T1w image were used. All ROIs were then propagated to the ASL derived CBF maps and three remaining DSC derived rCBV maps, which were all linked. The measurements within each lesion were thus derived from an identically sized ROI. The ROI average CBF and rCBV were obtained from which ratios between the tumor and NAWM were calculated by dividing the tumor ROI value by the NAWM ROI value. **Figure 1** shows an example of an ROI measurement.

### Data Analyses

Statistical analyses were performed using RStudio. Pearson’s correlation coefficient and 95% confidence intervals (CI) were calculated between the ASL-derived CBF ratio and each DSC-derived rCBV ratios for each lesion. A cluster bootstrapping approach was used to correct for the dependency of multiple lesions within the same patient. Correlations were assessed in all patients combined, and in subgroups of patients with 1) enhancing and 2) non-enhancing glioma; 3) brain metastasis; and 4) enhancing and 5) non-enhancing primary central nervous system lymphoma (PCNSL). All analyses were repeated after the exclusion of lesions with lesion-specific issues. Diagnostic accuracy for determining tumor progression was calculated as the area under the receiver operating characteristics (ROC) curve (AUC) at the patient level for the ASL-CBF ratio and each of the leakage corrected DSC-rCBV ratios (ISP, IBN). No ROC curves were created for the uncorrected rCBV measurements, as these are deemed clinically inferior to leakage-corrected rCBV.

## Results

### Patient and Lesion Characteristics

A total of 115 patients with 186 lesions were evaluated with both ASL and DSC-MRI. Eight lesions were excluded from further analysis due to severe motion artefacts or other technical issues. A total of 178 lesions were deemed eligible for further analysis. **Table 1** shows the patient characteristics in detail. All patients had undergone treatment (radiation and/or chemotherapy). Identified lesions were 80 enhancing glioma, 52 non-enhancing glioma, 31 enhancing metastases, 8 enhancing lymphoma, and 7 non-enhancing—residual lesions after treatment for lymphoma.

**Table 1:** Patient characteristics.

*Glioma*	
No. of patients	93
No. of lesions	132
Age in year (mean ± SD)	52.9 ± 12.8
Gender (male/female)	64/29
Enhancing lesions	80
Non-enhancing lesions	52
	
*Metastases*	
No. of patients	16
No. of lesions	31
Age in year (mean ± SD)	57.1 ± 12.8
Gender (male/female)	5/11
Enhancing lesions	31
Non-enhancing lesions	0
Primary tumor	Lung cancer 7 Breast cancer 3 Melanoma 2 Other 4
	
*Lymphoma*	
No. of patients	6
No. of lesions	15
Age in year (mean ± SD)	61.8 ± 13.4
Gender (male/female)	4/2
Enhancing lesions	8
Non-enhancing lesions	7

### Image Quality and Lesion-Specific Issues

Quality assessment identified 66 lesions with issues (**Table 2**): 63 with DSC-MRI and 50 with ASL; 47 lesions had both DSC-MRI and ASL issues. Lesion-specific issues included cortical lesion localization, the lesion being too small to measure, and signal loss in the lesion. Lesion signal loss was due to susceptibility artifacts (e.g., hemosiderin, melanin, and metallic surgical materials). The latter was more prominent with DSC-MRI (25 lesions) than with ASL (5 lesions). With ASL, there were 2 lesions outside the scanned field of view that was not observed with DSC-MRI.

**Table 2:** Lesion-specific issues.

	*No.*
**ASL-specific issues (total)**	**50**
Lesion in midline	3
Cortical localization	10
Too small to measure	29
Extensive white matter disease	1
Signal loss at tumor localization	5
Outside scanning range	2
Other issue	5
**DSC-specific issues (total)**	**63**
Lesion in midline	3
Cortical localization	10
Too small to measure	28
Extensive white matter disease	1
Signal loss at tumor localization	25
Outside scanning range	0
Other issue	4

PM Lesions can have more than one lesion specific issue.

### Correlation Between ASL-Derived CBF and DSC-Derived rCBV Ratios

Correlation coefficients and plots of ASL-derived CBF-ratios versus the leakage uncorrected and corrected DSC-derived rCBV-ratios (ISP, IBN) are shown in **Table 3** and **Supplementary Figure 1**, respectively.

**Table 3:** Correlation coefficients of lesion measurements.

**(A)**	**All lesions (n = 178)**	**Lesions without issues (n = 112)**
uncorrected DSC_rCBV ratio (ISP)	r = 0.67 (95% CI: 0.53–0.80)	r = 0.73 (95% CI: 0.60–0.86)
corrected DSC_rCBV ratio (ISP)	r = 0.78 (95% CI: 0.65–0.88)	r = 0.82 (95% CI: 0.68–0.90)
uncorrected DSC_rCBV ratio (IBN)	r = 0.60 (95% CI: 0.48–0.72)	r = 0.72 (95% CI: 0.62–0.80)
corrected DSC_rCBV ratio (IBN)	r = 0.72 (95% CI: 0.64–0.81)	r = 0.77 (95% CI: 0.69–0.86)

**(B)**	**Enhancing glioma (n = 80)**	**Enhancing glioma without issues (n = 41)**
uncorrected DSC_rCBV ratio (ISP)	r = 0.75 (95% CI: 0.60–0.86)	r = 0.79 (95% CI: 0.61–0.89)
corrected DSC_rCBV ratio (ISP)	r = 0.80 (95% CI: 0.66–0.90)	r = 0.83 (95% CI: 0.70–0.92)
uncorrected DSC_rCBV ratio (IBN)	r = 0.65 (95% CI: 0.54–0.77)	r = 0.67 (95% CI: 0.55–0.81)
corrected DSC_rCBV_ ratio (IBN)	r = 0.73 (95% CI: 0.64–0.85)	r = 0.69 (95% CI: 0.54–0.85)

**(C)**	**Non-enhancing glioma (n = 52)**	**Non-Enhancing glioma without issues (n = 47)**
uncorrected DSC_rCBV ratio (ISP)	r = 0.58 (95% CI: 0.34–0.77)	r = 0.54 (95% CI: 0.30–0.73)
corrected DSC_rCBV ratio (ISP)	r = 0.67 (95% CI: 0.42–0.83)	r = 0.66 (95% CI: 0.42–0.82)
uncorrected DSC_rCBV ratio (IBN)	r = 0.65 (95% CI: 0.18–0.85)	r = 0.71 (95% CI: 0.20–0.89)
corrected DSC_rCBV ratio (IBN)	r = 0.73 (95% CI: 0.30–0.89)	r = 0.78 (95% CI: 0.37–0.92)

**(D)**	**All metastasis (n = 31)**	**Metastasis without issues (n = 14)**
uncorrected DSC_rCBV ratio (ISP)	r = 0.26 (95% CI: −0.33–0.54)	r = 0.31 (95% CI: −0.01–0.65)
corrected DSC_rCBV ratio (ISP)	r = 0.32 (95% CI: 0.13–0.69)	r = 0.17 (95% CI: −0.16–0.86)
uncorrected DSC_rCBV ratio (IBN)	r = 0.14 (95% CI: −0.47–0.51)	r = 0.29 (95% CI: −0.04–0.67)
corrected DSC_rCBV ratio (IBN)	r = 0.40 (95% CI: −0.28–0.78)	r = 0.52 (95% CI: 0.28–0.88)

**(E)**	Enhancing lymphoma (n = 8)*	
uncorrected DSC_rCBV ratio (ISP)	r = −0.24 (95% CI: −0.81–0.56)	
corrected DSC_rCBV ratio (ISP)	r = −0.51 (95% CI: −0.90–0.29)	
uncorrected DSC_rCBV ratio (IBN)	r = −0.09 (95% CI: −0.75–0.65)	
corrected DSC_rCBV ratio (IBN)	r = 0.10 (95% CI: −0.65–0.75)	

**(F)**	Non-Enhancing lymphoma (n = 7)*	
uncorrected DSC_rCBV ratio (ISP)	r = −0.22 (95% CI: −0.83–0.64)	
corrected DSC_rCBV ratio (ISP)	r = 0.03 (95% CI: −0.74–0.76)	
uncorrected DSC_rCBV ratio (IBN)	r = −0.69 (95% CI: −0.95–0.14)	
corrected DSC_rCBV ratio (IBN)	r = −0.63 (95% CI: −0.94–0.23)	

Correlation coefficients of all lesions **(A)**, subsets of enhancing glioma **(B)**, non-enhancing glioma **(C)**, metastasis **(D)**, enhancing lymphoma **(E),** and non-enhancing lymphoma **(F)**. Coefficients shown in the middle column are based on all lesions (with and without lesion specific issues). Coefficients shown in the right column are based on subsets from which lesions with issues were excluded.

*Because of the very small subset size of enhancing lymphoma and non-enhancing lymphoma (<10), cluster bootstrapping was not possible.

A moderate (r = 0.60–0.67) correlation between the ASL-derived CBF versus DSC-derived rCBV ratios without leakage correction was found for all lesions combined, which improved to a strong (r = 0.72–0.78) correlation when leakage correction was applied (**Table 3** and **Supplementary Figure 1**). This improvement in correlation with leakage correction was also found in the subgroup analysis when lesions with issues were excluded (**Table 3** and **Supplementary Figure 1**). In the disease-specific subgroup analyses, a strong correlation (r = 0.65–0.80) was found in enhancing glioma (**Table 3** and **Supplementary Figure 1**), also after the exclusion of lesions with issues. In non-enhancing glioma, the correlation was lower (r = 0.58–0.73) (**Table 3** and **Supplementary Figure 1**). In brain metastasis, the correlation between ASL-derived CBF and DSC-derived rCBV was weak (r = 0.14–0.40). No significant correlations were found in the small (N <10) subgroups of enhancing and non-enhancing lymphoma.

### Diagnostic Accuracy of ASL-Derived CBF and DSC-Derived rCBV Ratios

**Figure 2:**
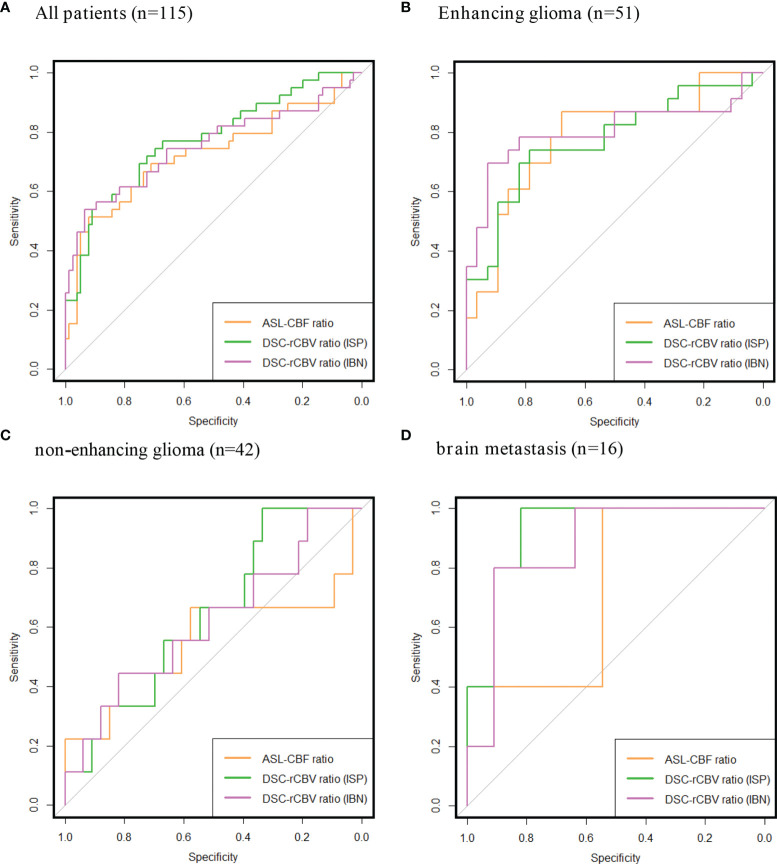
Receiver operating characteristics (ROCs) of ASL-CBF ratio, DSC-rCBV ratio Intellispace Portal (ISP) and DSC-rCBV ratio IB Neuro (IBN). All patients together **(A)**, subgroups of: enhancing glioma **(B)** non-enhancing glioma **(C)**, brain metastasis **(D)**. Area under the curves (AUC) and confidence intervals (CIs) are listed in **Table 4**.

The AUCs for diagnosing tumor progression for all patients combined, patients with enhancing glioma and patients with brain metastasis were within the same high range for both ASL-derived and DSC-derived leakage-corrected ratios (0.73–0.80) (**Table 4** and **Figure 2**). No AUCs were calculated for the lymphoma subgroups, given the insignificant number of patients in these subgroups. The AUC for enhancing glioma was higher than for non-enhancing glioma (AUC = 0.78–0.80 versus AUC = 0.56–0.62, respectively), both for ASL-derived CBF (AUC = 0.78) and for DSC-derived rCBV ratios (AUC = 0.77–0.80). In brain metastasis, the AUC was lower for ASL-derived CBF (AUC = 0.72) than for DSC-derived rCBV ratios (AUC = 0.87–0.93), although with wide and partly overlapping CIs.

**Table 4:** AUC values of diagnostic accuracy.

**(A) All patients (n = 115).**	
ASL-CBF ratio	0.73 (95% CI: 0.62–0.83)
corrected DSC-rCBV ratio (ISP)	0.78 (95% CI: 0.68–0.87)
corrected DSC-rCBV ratio (IBN)	0.75 (95% CI: 0.64–0.86)
**(B) Enhancing glioma (n = 51).**	
ASL-CBF	0.78 (95% CI: 0.65–0.91)
corrected DSC-rCBV ratio (ISP)	0.77 (95% CI: 0.60–0.91)
corrected DSC-rCBV ratio (IBN)	0.80 (95% CI: 0.66–0.94)
**(C) Non-enhancing glioma (n = 42).**	
ASL-CBF	0.56 (95% CI: 0.28–0.83)
corrected DSC-rCBV ratio (ISP)	0.64 (95% CI: 0.45–0.84)
corrected DSC-rCBV ratio (IBN)	0.62 (95% CI: 0.39–0.84)
**(D) Metastasis (n = 16).**	
ASL-CBF	0.72 (95% CI: 0.44–1.00)
corrected DSC-rCBV ratio (ISP)	0.93 (95% CI: 0.80–1.00)
corrected DSC-rCBV ratio (IBN)	0.87 (95% CI: 0.6–1.00)

**Table 4** shows the AUC of the ROCs with a 95% CI. Diagnostic accuracy was assessed for all patients together **(A)** and for subgroups of enhancing glioma **(B)**, non-enhancing glioma **(C),** and brain metastasis patients **(D)**.

## Discussion

In this retrospective study, we assessed the feasibility of using ASL perfusion imaging as an alternative to DSC perfusion imaging in different types of brain tumors at 3T MRI.

We found a high correlation between ASL and DSC derived perfusion parameters in enhancing glioma. In non-enhancing glioma and metastases, the correlation was much lower. There was no correlation in either enhancing or non-enhancing lymphoma, which could be explained by the small sample size and/or by the known lower perfusion of PCNSL (25). These findings suggest that CBF and rCBV provide a similar measure of tumor vascularity, in particular in enhancing glioma. Even though the parameters obtained from both perfusion techniques (CBF from ASL and rCBV from DSC) differ due to different diffusion behaviors of GBCA and water molecules in the tissue (26), several studies have shown that CBF derived from DSC has a similar diagnostic value as that of rCBV (which is considered the technically more robust perfusion metric) in distinguishing benign from malignant tumors and for tumor grading (14, 27, 28), so it could be expected that those perfusion parameters from ASL and DSC are also correlated. Across all subgroups (except enhancing lymphoma), correlation coefficients improved when leakage correction was applied. This finding supports the notion that ASL-derived CBF—not being affected by the T1 and T2* leakage effects from which DSC-MRI suffers—provides a more “true” measure of tumor perfusion (29). The fact that correlations were not as strong in non-enhancing glioma and brain metastasis may be a reflection of underlying pathophysiology, and in particular, the fact that both have lower vascularization than enhancing glioma (13, 30). Another explanation could be that T2*w DSC is less sensitive to tissue with lower microvasculature (31). As more lesions showed signal loss due to susceptibility artifacts with DSC than with ASL, this is an important consideration for choice of technique in patients with tumors with a high probability of susceptibility artifacts, such as lesions that are prone to hemorrhage, or in the postsurgical or post-therapeutic setting.

From a clinical perspective, it is particularly relevant to find that the diagnostic accuracy of ASL and DSC are within the same high range, particularly in enhancing glioma. For brain metastasis, the diagnostic accuracy was lower for ASL than for DSC, although the CIs were partly overlapping and thus formally not significantly different. This suggests that either technique may be used, but that care must be taken in interpreting the measured ratios as these may not be comparable across techniques. Additionally, note that there is some uncertainty related to establishing the final diagnosis. In most cases, no histopathological confirmation was available, and clinical and radiological follow-up were relied upon to determine the most likely diagnosis of tumor progression versus pseudoprogression, stable disease, and response.

There are few studies in the published literature examining the diagnostic accuracy of ASL perfusion versus DSC. In a study by Ata et al. (2016), a comparison of these techniques was carried out in 27 patients with primary and metastatic brain tumors, and showed similar diagnostic value, with sensitivities of 88% (ASL) and 94% (DSC). Both techniques showed a specificity of 100% (14). A study by Morana et al. (2018) analyzed the blood flow of astrocytic tumors in 37 pediatric patients using ASL and DSC, and found that ASL provided comparable results to DSC, allowing distinction between high- and low-grade astrocytic tumors with AUCs of 0.96 for both ASL and DSC (32). Manning et al. (2020) compared non-contrast and contrast MRI perfusion in 32 patients with glioma for the differential diagnosis of tumor progression and pseudoprogression after treatment, finding that both techniques had nearly equivalent performance with an AUC of 0.95 for ASL and an AUC of 0.89 for DSC, and that ASL had reduced sensitivity to susceptibility artifacts (12). Thus, all these studies revealed a significant correlation between ASL and DSC parameters, but the small numbers of included patients did not allow the results to be applied in practice. Additionally, we did not find studies comparing ASL and DSC in brain metastasis and in PCNSL. The current study examined a larger group of patients with different entities, providing evidence for the potential usefulness of ASL perfusion in clinical practice.

While ASL and DSC showed comparable diagnostic accuracy, ASL showed fewer lesion-specific issues than DSC. In particular, the presence of susceptibility artifacts causing signal loss at the site of the lesion is a real disadvantage of DSC-MRI (occurring in 25 of 178 lesions). Susceptibility artifacts are commonly seen due to localization near large vessels, the skull base or the scalp, and in the presence of hemorrhage after surgery or radio-/chemotherapy in primary and metastatic brain tumors (33, 34). Conversely, only a small number of lesions (2 of 178 lesions) suffered from an ASL-specific issue, namely its field of view: sometimes the lower portion of the cerebellum is not imaged with ASL, rendering the technique less suitable for very inferiorly located lesions (34).

The following limitations should be noted: Our study was retrospective, but overall, we included a large dataset of 115 patients comprising 178 lesions. Also, since the study was conducted at 3T MRI, we cannot determine its applicability at 1.5T. Results cannot be simply assumed to apply to lower field strengths, in particular because ratios were calculated relying on the normal appearing white matter, the low ASL signal of which becomes more problematic and unreliable at lower field strengths. A further limitation is the way the final diagnosis was established. In most cases, histopathological confirmation was lacking, so the diagnosis was made clinically and radiologically, taking the course of symptoms and imaging abnormalities over time into account. While reliable and reproducible parameters are required during follow-up (especially when histopathology is lacking) for a certain diagnosis, these are not always available in routine clinical practice (35).

In conclusion, we found that ASL and DSC-MRI have more or less the same diagnostic accuracy. Our findings suggest that ASL can be used as an alternative to DSC-MRI to measure perfusion in enhancing and non-enhancing gliomas and brain metastasis at 3T. For lymphoma, this should be further investigated in a larger group.

## Data Availability Statement

The datasets presented in this article are not readily available because informed consent for sharing raw MRI data has not been provided. The original measurements and analysis scripts are available upon request. Requests to access the datasets should be directed to MS, marion.smits@erasmusmc.nl.

## Ethics Statement

The studies involving human participants were reviewed and approved by the Erasmus MC Medical Ethics Committee. The patients/participants provided their written informed consent to participate in this study.

## Author Contributions

AL and WT share first authorship and contributed equally. AL: data collection and writing. WT: study design, data collection, data analysis, and writing. MB: correcting draft from clinical perspective. EW: correcting draft from technical perspective. MS: study design, draft corrections, and daily supervision. All authors listed have made a substantial, direct, and intellectual contribution to the work and approved it for publication.

## Funding

WT is funded by “Leading the Change” (80-85009-98-2008-NVvR). EW is funded by a “Veni Vernieuwingsimpuls” from the Dutch Research Council entitled “Food for thought: Oxygen delivery to the brain”, grant number 91619121.

## Conflict of Interest

MS declares speaker fees from GE Healthcare (paid to institution).

The remaining authors declare that the research was conducted in the absence of any commercial or financial relationships that could be construed as a potential conflict of interest.

The handling Editor declared a past co-authorship with several of the authors MS, EW.

## Publisher’s Note

All claims expressed in this article are solely those of the authors and do not necessarily represent those of their affiliated organizations, or those of the publisher, the editors and the reviewers. Any product that may be evaluated in this article, or claim that may be made by its manufacturer, is not guaranteed or endorsed by the publisher.
