# Supplementary material for: Diagnostic Accuracy of Arterial Spin Labeling in Comparison With Dynamic Susceptibility Contrast-Enhanced Perfusion for Brain Tumor Surveillance at 3T MRI
Source: Front Oncol. 2022 May 20;12:849657. doi: 10.3389/fonc.2022.849657 (PMC9163566; doi:10.3389/fonc.2022.849657)
Supplement: Supplementary file 1 [file DataSheet_1.docx]

**Supplementary Figure 1**

**(A)** all lesions (n = 178)

| ASL-CBF ratios versus DSC-rCBV leakage uncorrected ratios (ISP) | ASL-CBF ratios versus DSC-rCBV leakage corrected ratios (ISP) |
| --- | --- |
| 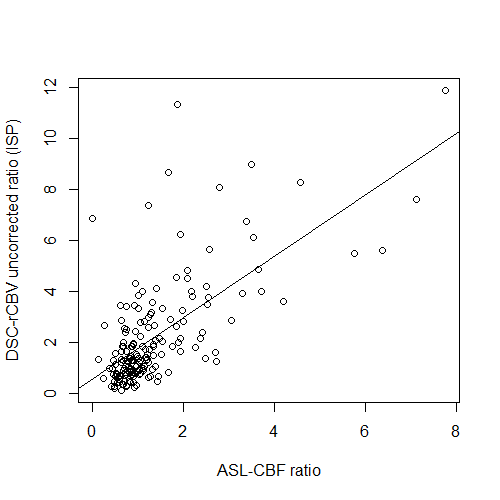 | 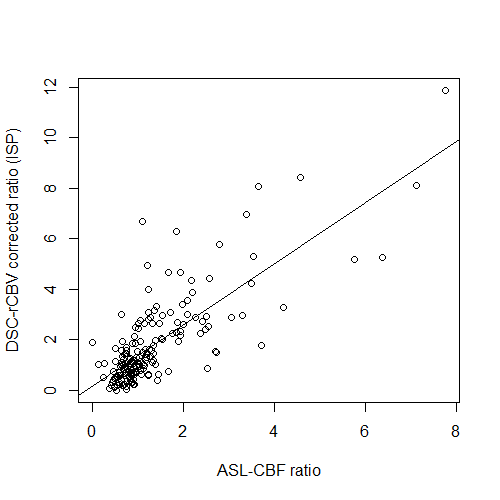 |
| r = 0.67 (95% CI: 0.58-0.75) | r = 0.78 (95% CI: 0.72-0.83) |
| ASL-CBF ratios versus DSC-rCBV leakage uncorrected ratios (IBN) | ASL-CBF ratios versus DSC-rCBV leakage corrected ratios (IBN) |
| 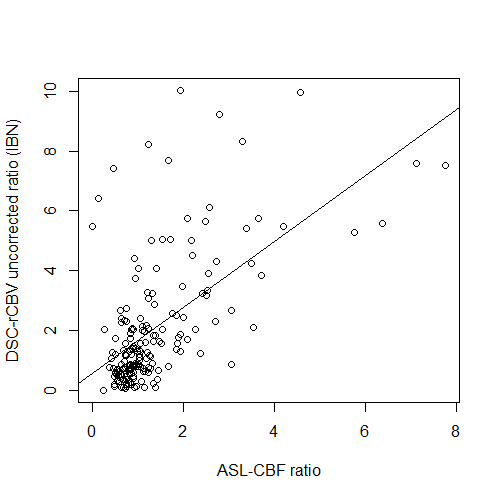 | 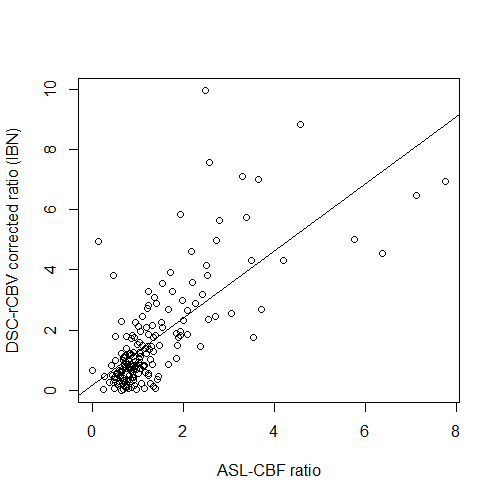 |
| r = 0.60 (95% CI: 0.50-0.69) | r = 0.72 (95% CI: 0.65-0.79) |

**(B)** enhancing glioma (n = 80)

| ASL-CBF ratios versus DSC-rCBV leakage uncorrected ratios (ISP) | ASL-CBF ratios versus DSC-rCBV leakage corrected ratios (ISP) |
| --- | --- |
| 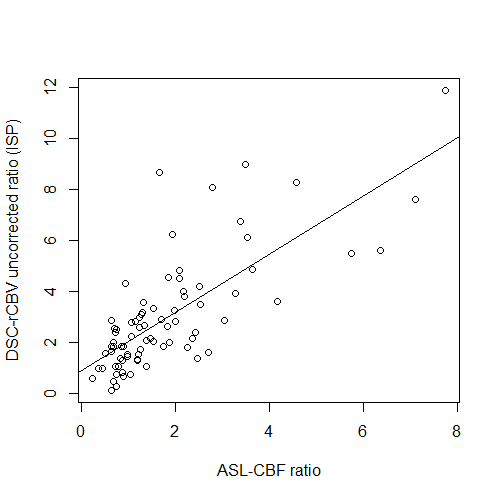 | 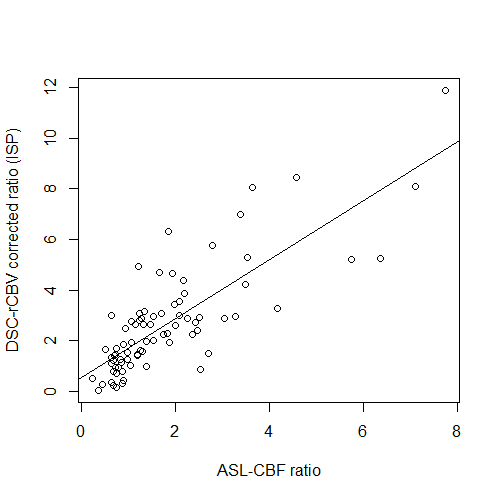 |
| r = 0.75 (95% CI: 0.63-0.83) | r = 0.80 (95% CI: 0.71-0.87) |
| ASL-CBF ratios versus DSC-rCBV leakage uncorrected ratios (IBN) | ASL-CBF ratios versus DSC-rCBV leakage corrected ratios (IBN) |
| 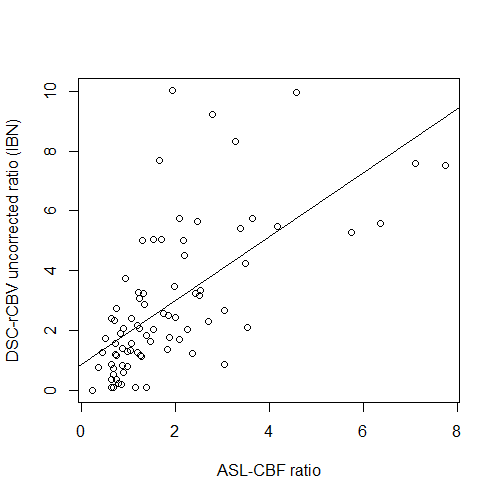 | 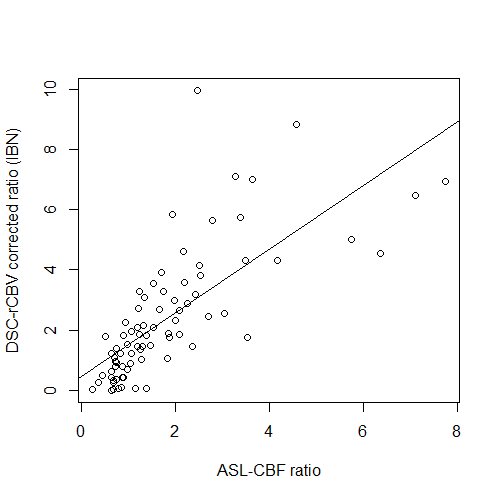 |
| r = 0.65 (95% CI: 0.51-0.76) | r = 0.73 (95% CI: 0.61-0.82) |

**(C)** non-enhancing glioma (n = 52)

| ASL-CBF ratios versus DSC-rCBV leakage uncorrected ratios (ISP) | ASL-CBF ratios versus DSC-rCBV leakage corrected ratios (ISP) |
| --- | --- |
| 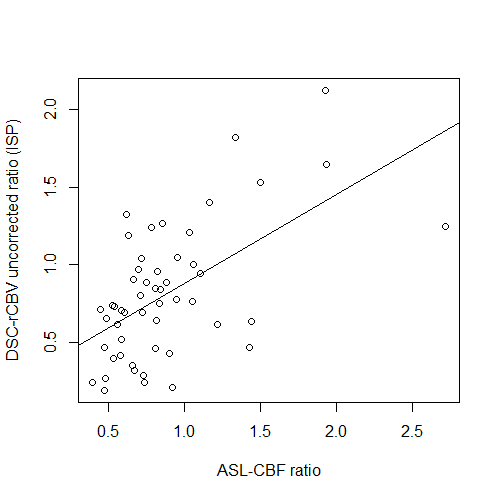 | 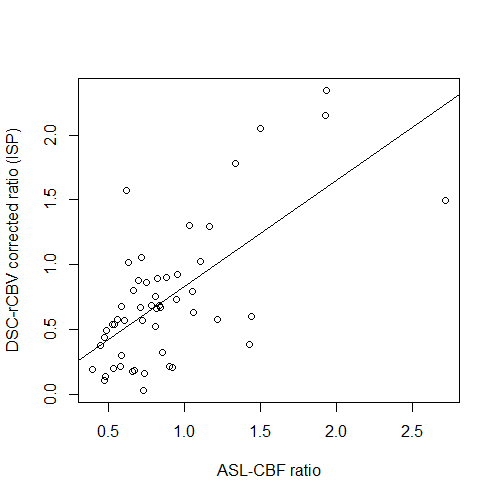 |
| r = 0.58 (95% CI: 0.37-0.74) | r = 0.67 (95% CI: 0.48-0.80) |
| ASL-CBF ratios versus DSC-rCBV leakage uncorrected ratios (IBN) | ASL-CBF ratios versus DSC-rCBV leakage corrected ratios (IBN) |
| 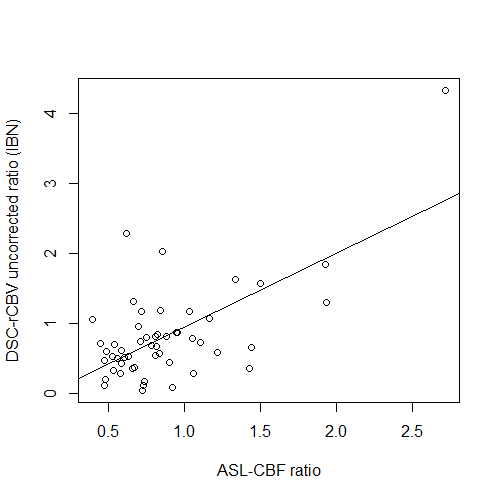 | 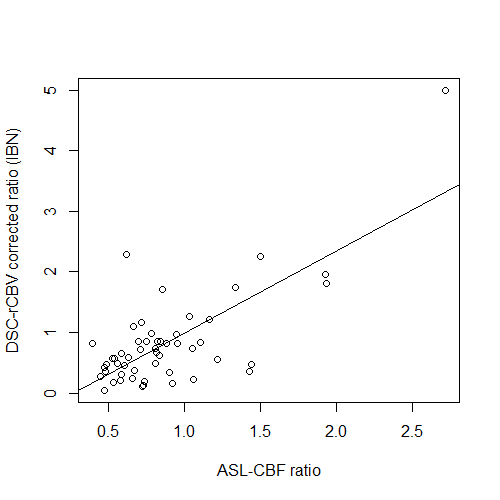 |
| r = 0.65 (95% CI: 0.46-0.79) | r = 0.73 (95% CI: 0.57-0.84) |

(**D)** metastasis (n = 31)

| ASL-CBF ratios versus DSC-rCBV leakage uncorrected ratios (ISP) | ASL-CBF ratios versus DSC-rCBV leakage corrected ratios (ISP) |
| --- | --- |
| 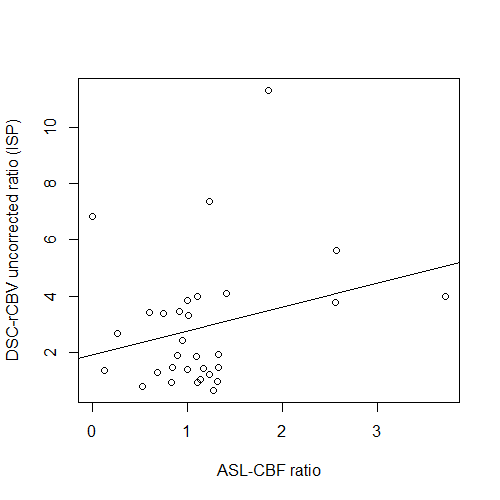 | 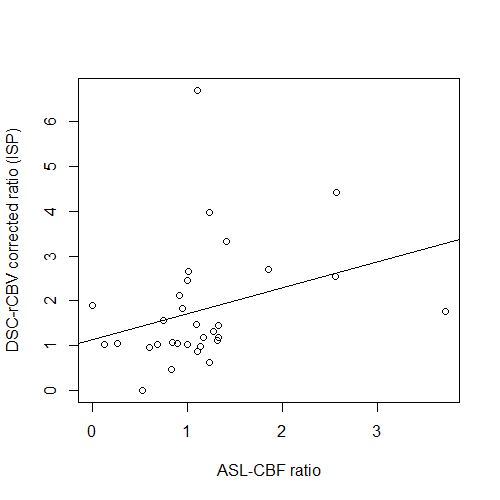 |
| r = 0.26 (95% CI: -0.1-0.57) | r = 0.32 (95% CI: -0.04-0.60) |
| ASL-CBF ratios versus DSC-rCBV leakage uncorrected ratios (IBN) | ASL-CBF ratios versus DSC-rCBV leakage corrected ratios (IBN) |
| 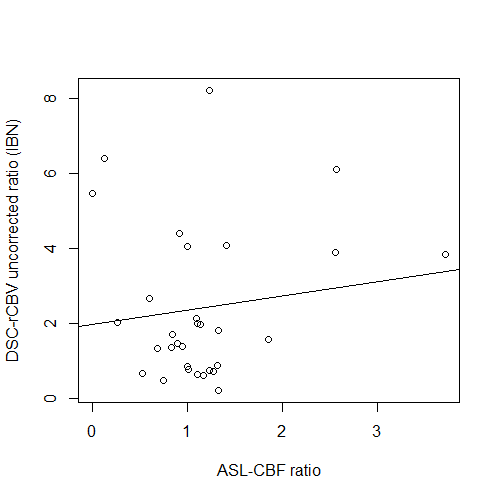 | 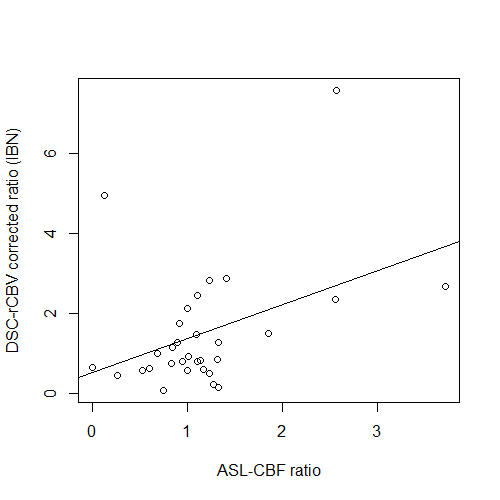 |
| r = 0.14 (95% CI: -0.23-0.47) | r = 0.40 (95% CI: 0.06-0.66) |

**Supplementary figure 1** shows correlation plots of ASL-CBF versus DSC-rCBV both uncorrected for leakage and corrected for leakage using two software packages for DSC, Intellispace Portal (ISP) and IB Neuro (IBN). Correlation plots are calculated for all lesions together **(A)** and for subgroups of enhancing glioma **(B)**, non-enhancing glioma **(C)** and brain metastasis **(D)**.
